# Estimation of gait parameters using leg velocity for amputee population

**DOI:** 10.1371/journal.pone.0266726

**Published:** 2022-05-13

**Authors:** Zohaib Aftab, Rizwan Shad

**Affiliations:** 1 Department of Mechanical Engineering, Faculty of Engineering, University of Central Punjab, Lahore, Pakistan; 2 Human-centered robotics lab, National Center of Robotics and Automation (NCRA), Rawalpindi, Pakistan; Universiti Sains Malaysia, MALAYSIA

## Abstract

Quantification of key gait parameters plays an important role in assessing gait deficits in clinical research. Gait parameter estimation using lower-limb kinematics (mainly leg velocity data) has shown promise but lacks validation for the amputee population. The aim of this study is to assess the accuracy of lower-leg angular velocity to predict key gait events (toe-off and heel strike) and associated temporal parameters for the amputee population. An open data set of reflexive markers during treadmill walking from 10 subjects with unilateral transfemoral amputation was used. A rule-based dual-minima algorithm was developed to detect the landmarks in the shank velocity signal indicating toe-off and heel strike events. Four temporal gait parameters were also estimated (step time, stride time, stance and swing duration). These predictions were compared against the force platform data for 3000 walking cycles from 239 walking trials. Considerable accuracy was achieved for the HS event as well as for step and stride timings, with mean errors ranging from 0 to -13ms. The TO prediction exhibited a larger error with its mean ranging from 35-81ms. The algorithm consistently predicted the TO earlier than the actual event, resulting in prediction errors in stance and swing timings. Significant differences were found between the prediction for sound and prosthetic legs, with better TO accuracy on the prosthetic side. The prediction accuracy also appeared to improve with the subjects’ mobility level (K-level). In conclusion, the leg velocity profile, coupled with the dual-minima algorithm, can predict temporal parameters for the transfemoral amputee population with varying degrees of accuracy.

## Introduction

Gait analysis plays an important role in detecting and characterizing various diseases and amputations. Accurate gait analysis requires the determination of key gait events of heel strike (HS) and toe-off (TO) for gait segmentation into swing and stance phases [1]. Gait event prediction also leads to the estimation of several temporal parameters (such as step timings, phase durations) used to assess overall gait quality and symmetry in individuals.

In a conventional clinical setting, a visual or video examination of gait by expert observers can give a basic functional assessment of gait. To quantify these events, kinetic data obtained from force platforms and pressure mats are considered a gold standard. This method uses a simple threshold on the force/pressure to discriminate between swing and stance phases. Due to high cost and space constraints, force platforms are not available everywhere, particularly in clinics and outdoor settings. Moreover, it only detects events from a limited number of steps (usually one or two) depending upon the number of force platforms. Cheaper kinetic alternatives have been investigated (e.g. using pressure insoles [2]) but with poor repeatability of results for prosthetic users.

An alternative to this method is using algorithm-based event detection using optoelectronic (marker) data [3, 4] or inertial sensors [5, 6]. These methods rely on leg or foot kinematics and rule-based algorithms to estimate gait events. Numerous studies have focused on validating this method for TO and HS detection for healthy subjects [5, 7–13] as well as for pathological gait [3, 14–18]. A few studies have included amputee subjects [19, 20], but have failed to establish its validity due to a small sample size or a non-standard reference method.

Several rule-based algorithms have been developed to identify observable features in the velocity/acceleration data of body segments. Aminian et. al. [21] first reported the coincidence of the minima in the shank sagittal-plane angular velocity with HS and TO events. This algorithm is a popular choice among researchers, who have used this method to predict gait events/parameters for diverse subject populations with a reasonable degree of accuracy [7, 8, 12, 16, 19, 22, 23]. However, no such study focused on amputee data, except [19] which included data from a single subject. Hence, the accuracy of this method in predicting gait events and parameters for amputees has not been established in the literature.

An amputee’s gait deviates from a healthy gait due to several reasons including muscle weakness, pain, and decreased confidence. In addition, the type of prosthesis and its fit/alignment also affects amputee gait pattern [24]. Together, these factors often lead to an altered gait pattern involving phenomena such as vaulting, circumduction, and foot slap [25]. These factors could adversely affect the prediction accuracy of this algorithm and there is a need to evaluate it using large data set of people with amputations.

The aim of this study is to compare the accuracy of gait event prediction using the sagittal-plane shank angular velocity against the force platform data as the reference. A secondary objective is to illustrate the effect of subjects’ walking ability and speed on the prediction accuracy. A published data set of amputee subjects by Hood et. al. [26] is used for this purpose containing optoelectronic and force plate data for transfemoral amputees. To the authors’ knowledge, this is the largest published data set of its kind.

## Materials and methods

A data set consisting of marker and force plate data of 18 individuals with unilateral transfemoral amputation was recently published [26]. Raw data from this data set was synthesized and used in this study.

### Data collection

The data set includes kinematics and kinetics collected while walking on an instrumented treadmill at five different speeds. The raw data recorded consisted of three-dimensional trajectories of 61 cutaneous reflective markers spread over the whole body, and the kinetics collected from the force plates. Marker trajectory data was collected using a 10-camera Vicon system (Vicon Motion Systems Ltd; Oxford, UK) at 200 Hz and the ground reaction force data using a split-belt instrumented treadmill (Bertec Co; Columbus, OH) at 1000 Hz. Reflective markers (14-mm diameter, 2-mm base) were placed on the subject following a modified Plug-in-Gait Model.

A total of 18 subjects with an above-knee amputation participated in this study. All subjects had received a unilateral above-knee amputation at least one year prior to the enrollment, had used a prescribed prosthesis for at least six months and a minimum of 3 hours a day. All but one subject used prostheses with microprocessor-controlled knee joint. All subjects employed a passive ankle joint.

Subjects were divided into two groups based on their comfortable walking speed and reliance on the handrails. They were categorized as either K-level 2 or K-level 3 on the Medicare functional classification level (MFCL) [27]. If a subject required the assistance of handrails for any speed above 0.8 m/s or their maximum walking speed was 0.8 m/s, they were assigned to the K2 group. This group of subjects walked at five different speeds of [0.4, 0.5, 0.6, 0.7, 0.8m/s]. Likewise, if subjects could walk at speeds upto 1.2 m/s without using handrails, they were assigned to the K3 group, and they walked at the speeds of [0.6, 0.8, 1.0, 1.2, 1.4 m/s].

It is the most comprehensive gait data set available for prosthesis users, which provides force platform data for all steps taken during a trial. The original study contained an equal number of subjects in both groups. However, for this study, we selected a subset of the subjects that did not use handrails to avoid the effect of secondary support on the gait pattern. Ten out of 18 subjects fulfilled this criterion (including three K-level 2 and seven K-level 3 subjects) and were subsequently analyzed. These subjects are listed in Table 1. All ten subjects in this study had undergone amputation as a result of trauma.

**Table 1:** List of subjects for whom the walking data is used in this study. Complete details on amputation can be found in [26].

Subject	Age	Gender	K-level	Number of trials in the analysis
TF01*	26	Male	K3	21
TF05	72	Male	K2	25
TF07**	49	Male	K3	22
TF08	42	Male	K3	25
TF09	65	Male	K2	25
TF11**	51	Male	K3	23
TF12**	59	Male	K2	23
TF16	36	Male	K3	25
TF17	38	Male	K3	25
TF19	30	Female	K3	25
Total trials				239

Every individual is labeled as ‘TFxx’ where (TF) is noted for ‘Transfemoral Amputation’ and ‘xx’ is an assigned identification number. The data set reported four to five walking trials per speed, with a few exceptions, resulting in a total of 246 walking trials for the ten subjects. However, after careful observation of the force platform and marker profiles, some trials were discarded due to incomplete or erroneous data. This resulted in a total of 239 trials for final analysis.

### Estimation of leg velocity signal from marker data

**Fig 1:**
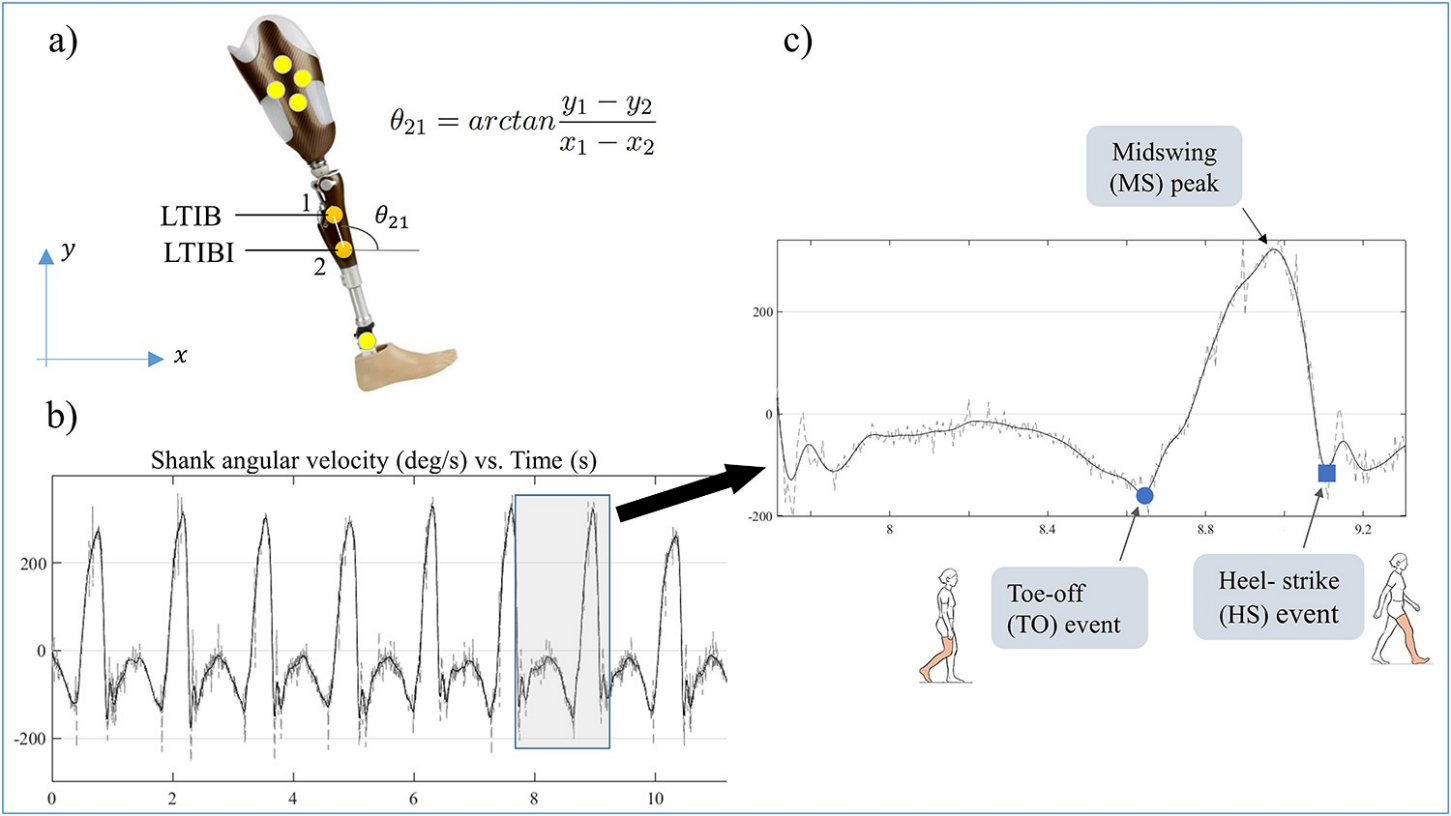
a): Placement of tibia markers for leg orientation and velocity calculation, b) A typical shank velocity signal with raw (grey) and filtered (black) data, c) Enlarged view of one gait cycle from the velocity signal. The algorithm starts with the detection of the largest positive peaks in the signal (marked as MS) which define the intervals for gait events. TO is identified as the last negative peak (or minima) just before the MS while HS is defined as the negative peak just after the MS.

The raw data contained the .c3d files for the marker trajectories and marked events, which were extracted using an open-source motion analyzer software MOKKA (Motion Kinematic and Kinetic Analyzer [28]). For each trial, the data were exported into a .csv file and read in Matlab to calculate lower-leg angular velocities from the coordinates of tibia markers. Two tibia markers on each leg (namely LTIB, LTIBI, RTIB, RTIBI indicated in Fig 1) were used using the method presented by Winter [29]. The method uses two markers in line with the bone axis and calculates the orientation of the segment using Eq (1). The velocity is simply the time derivative of the segment orientation.

$$\begin{array}{c} \theta_{21}=arctan\frac{y_{1}-y_{2}}{x_{1}-x_{2}} \end{array}$$ (1)

The raw marker data was collected at 200Hz, which is subject to a lot of noise due to soft tissue artifacts. In order to reduce the noise in the resulting angular velocity signal, a low pass filter was designed and implemented. For this purpose, the frequency spectrum and the Nyquist frequency of the signal for all subjects were analyzed. A cut-off frequency of 4 Hz was chosen, which resulted in negligible loss of data and time-shift of the signal (c.f. Fig 1).

### Algorithm

Toe-off and heel strike events are predicted using the dual-minima approach [23]. It starts with the detection of all the largest positive peaks in the velocity signal, which are marked as midswing (MS) events (c.f. Fig 1). Each positive peak is accompanied by two negative peaks (or minima) on either side indicating the reversal of leg velocity direction. The negative peak (NP) preceding the MS is marked as the toe-off event, while the one after is marked as the heel strike. The implementation of the algorithm is carried out in Matlab.

For each walking cycle, the timings for the TO and HS events obtained by this algorithm are compared against the force platform-based timings provided in the data set. The errors (eTO, eHS) are calculated by taking the difference between predicted and actual events. The error is positive when the predicted event precedes the actual event and vice versa. Furthermore, using the events information, four temporal gait parameters (step time, stride time, stance and, swing phase durations) are also computed. Only complete gait cycles (defined as HS-HS for the same leg) were analyzed while the half-cycles at the start and end of the trials were discarded. This resulted in around 3000 complete gait cycles from the 239 trials.

## Results

Trial averages of TO and HS events as well as four gait parameters are compared using the descriptive statistics of mean error (ME), mean absolute error (MAE), and standard deviation. A Matlab file containing this data is provided as a supplement to this article.

Table 2 summarizes the error values for both events, separately for both legs. As a whole, HS error was smaller compared to TO error on sound (eHS: -5.48 ms) as well on prosthetic side (eHS: -13.15 ms). HS mean error was slightly negative indicating late detection by the algorithm. eTO on the sound side was the largest of all errors (ME of 80.77ms). The predicted TO was earlier than the actual event for 99 percent of the steps, albeit with a smaller magnitude on the prosthetic side (ME of 34.7ms) compared to the sound side. For both events, statistically significant differences were found between legs (p <.001, Wilcoxon signed-rank test).

**Table 2:** Mean error values for heel strike and toe-off events.

Gait event	Leg	Mean Error (ME) in milliseconds	Statistical Significance
Heel strike (HS)	Sound	-5.48 (±19.7)	p<.001
	Prosthetic	-13.15 (±37.5)	
Toe-off (TO)	Sound	80.77 (±21.4)	p<.001
	Prosthetic	34.70 (±16.8)	

**Fig 2:**
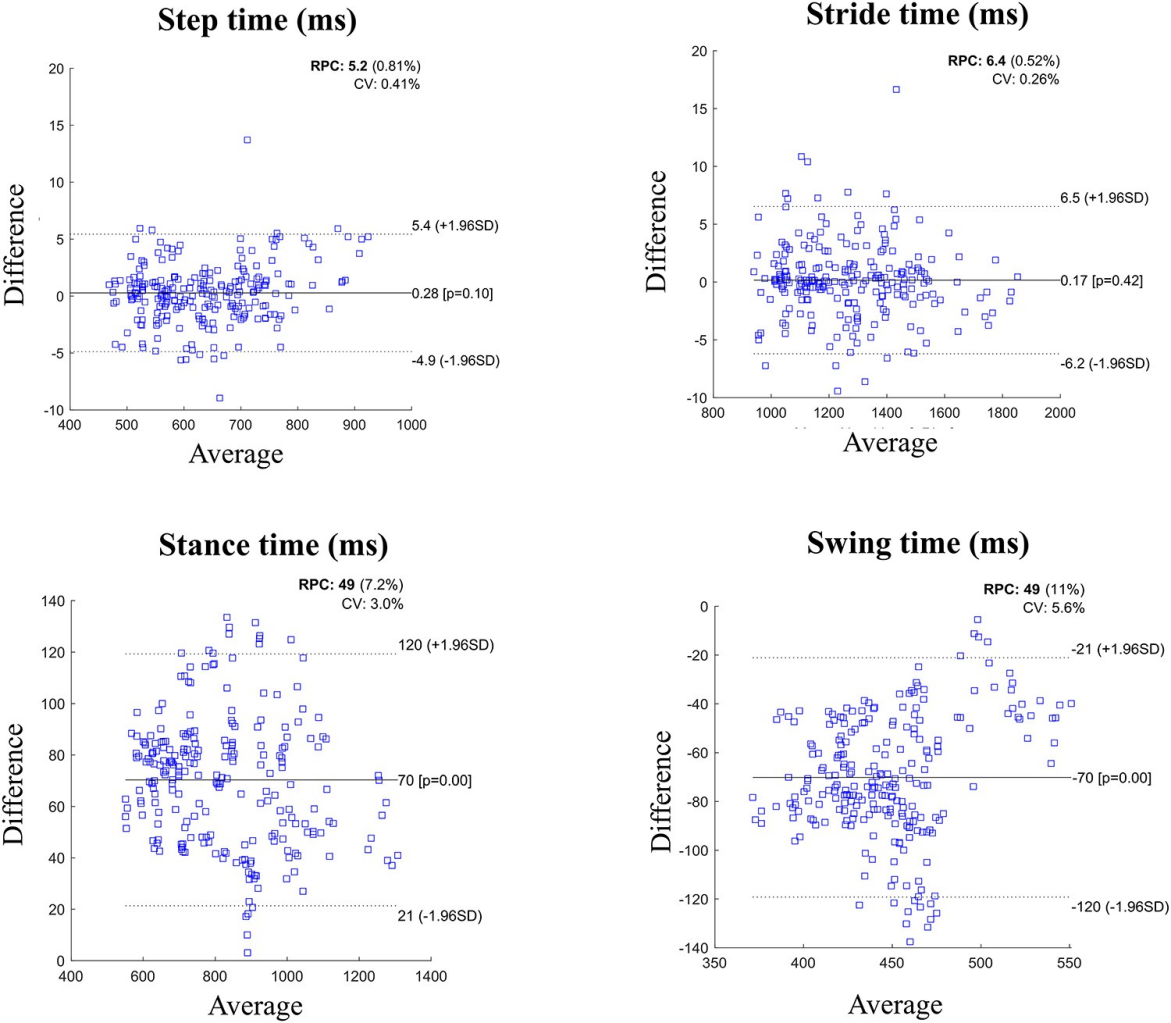
Bland Altman plots illustrating the agreement of four temporal parameters calculated using the algorithm-based method and those derived from the force-plate data. The average difference is specified by a solid line while limits of agreement (± 1.96SD) are represented by dotted lines. The figure files have also been provided in supplement to this article. RPC: Reproducibility co-efficient. CV: Coefficient of variation.

Fig 2 shows the Bland-Altman (BA) plots illustrating the agreement between predicted and actual values for the four temporal parameters. Mean difference for step and stride times (top row) was almost 0ms with a small standard deviation of the order of 5–6ms. For the stance and swing times (bottom row), the mean difference was larger. The stance duration calculated using the algorithm-based gait events was under-estimated by 70ms on average. Since a gait cycle is the sum of stance and swing durations, the swing duration was over-estimated by the same amount (indicated by the mean of -70ms).

Table 3 shows the mean absolute error values for gait events and parameters. Mean absolute HS error value was of the order of 17–40ms. As a result, the absolute step and stride time errors were also non-negligible (~17–44 ms range). Mean absolute TO error was larger with the same order as in Table 2 due to predominantly positive TO error values. Consequently, the stance and swing durations also had larger errors of the order of ~55–91ms.

**Table 3:** Absolute error values for gait events and parameters.

Gait event/ parameter	Leg	Mean Absolute Error (MAE) in milliseconds
Heel strike (HS)	Sound	17.24 (±14.3)
	Prosthetic	40.61 (±20.6)
Toe-off (TO)	Sound	80.87 (±21.2)
	Prosthetic	36.28 (±14.3)
Step time	-	44.38 (±23.6)
Stride time	Sound	17.03 (±14.05)
	Prosthetic	33.02 (±23.6)
Stance time	Sound	90.81 (±26.2)
	Prosthetic	55.60 (±33.9)
Swing time	Sound	90.97 (±26.8)
	Prosthetic	54.97 (±33.7)

### Differences by functional classification level

To observe the effect of subject’s functional classification level on the prediction error, separate error magnitudes were calculated for the K2 and K3 subjects. A Mann-Whitney U test was also performed to identify any significant differences between the groups.

**Fig 3:**
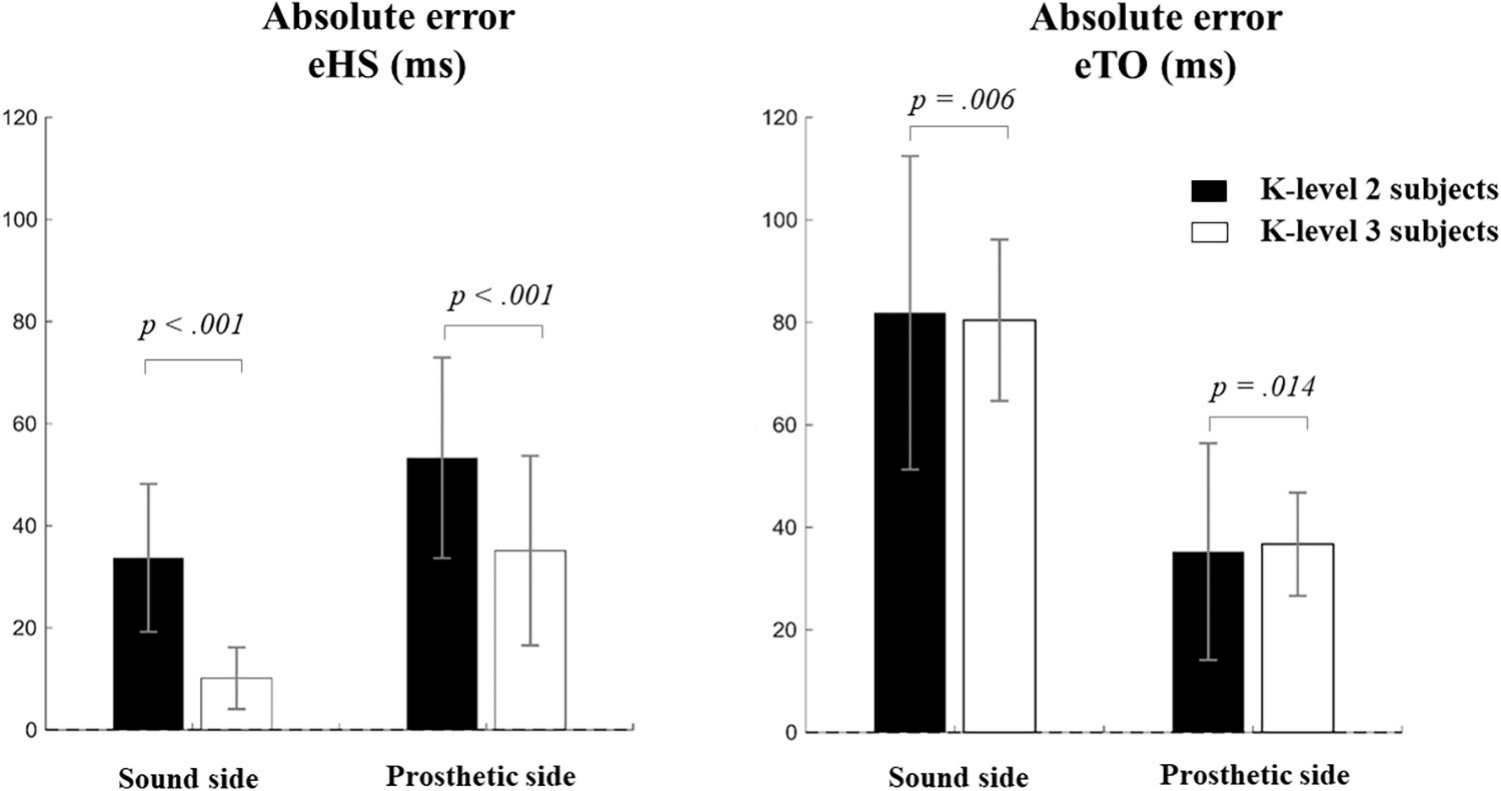
Heel-strike (left) and Toe-off (right) absolute error values separated by subject groups: Black bars for K-level 2 subjects and white bars for K-level 3. P-values are indicated for the Mann-Whitney U test comparing the two subject groups. In general, K3 subjects showed smaller error values and/or standard deviations.

A comparison of results is presented in Fig 3. The mean absolute error in the HS estimation was visibly smaller for the K3 subjects than for K2 individuals for both legs. However, the TO error showed a very small difference between groups, albeit with smaller standard deviation values for the K3 group.

A Mann-Whitney U test indicated statistically significant differences between both groups in all cases, with *p < .001* for HS errors while *p = .006* and *p = .014* for the TO errors on the sound and prosthetic sides respectively.

### Effect of walking speed

**Fig 4:**
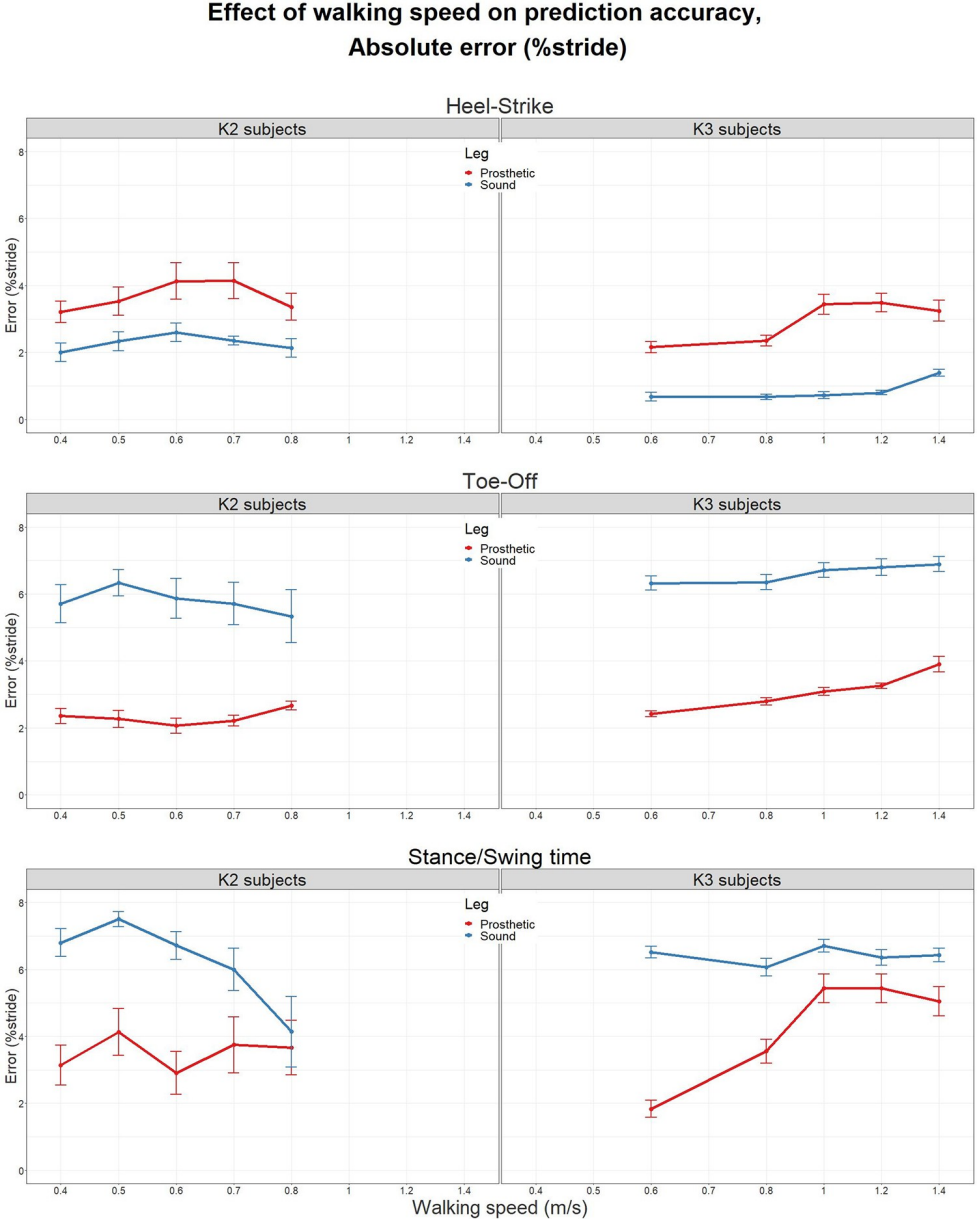
Speed-wise heel-strike (top), toe-off (middle), and stance/swing time (bottom) absolute error values, further separated by subject groups (left: K-level 2 subjects and right: K-level 3 subjects) and leg side (blue: sound, red: prosthetic).

Finally, to observe if the walking speed affected the prediction accuracy, mean absolute error values were plotted against the walking speed for gait events as well as stance/swing time (c.f. Fig 4). The error values are normalized by stride duration for a meaningful comparison across speeds. Errors for K2 subjects are plotted in the left panel while K3 subjects are plotted on the right and are further separated by leg on each plot.

While HS error values remained comfortably within the 5% of stride duration at all speeds, the magnitude of error for TO and swing/stance time estimation went up to 8% on the sound leg. Repeated measures ANOVA did not reveal any within-subject differences of error magnitude against walking speed for K2 subjects. However, for K3 subjects, all three parameters showed strong correlation with speed, especially on the prosthetic side.

## Discussion

This is one of the largest studies for any population comparing the kinematics-based gait event prediction with the force platform data.

A key finding from this study is the consistent early prediction of the toe-off event in 99% of the steps for both legs. This means that the actual toe-off takes place after the 1^st^ negative peak or minima of the velocity signal and points toward an inherent limitation of this algorithm. Group comparisons indicated that the prediction accuracy significantly improved for K3 subjects (Fig 3, right panel). However, the magnitude of error was still of the order of 40–80ms on average. Some studies have proposed the point of zero-crossing as TO event (e.g. [30]). This is the point where the signal crosses from negative to positive velocity and is located right after the negative peak (c.f. Fig 1). However, observation of our velocity signals from this data set does not support this view. We postulate that the actual TO event occurs after the negative peak (NP) but before the zero-crossing (ZC) of the velocity signal, hence yielding a narrow NP-ZC zone. Future studies should focus on this zone for accurate prediction of the TO event.

On the other hand, the HS prediction was slightly late in most cases, as indicated by negative ME values in Table 2. This means that the actual heel-strike occurs before the 2^nd^ negative peak. However, the error magnitude was small compared to the TO error, resulting in a better estimation of step and stride duration (Fig 2).

Group comparison in Fig 3 indicated that the error magnitude was larger for K2 subject group than for K3 subjects, particularly on the prosthetic side. The difference was also statistically significant. Literature studies estimating the HS event for healthy subjects using the same algorithm have reported absolute HS errors in the range of 7–24ms [7, 8, 16]. In this study, the K2 subjects exhibited absolute error values of over 50ms on average (c.f. Fig 3). From the clinical viewpoint, it could indicate that K2 subjects manifest greater gait deviations from the healthy template. This warrants precaution when using this method for subjects classified as ‘limited’ community ambulators on the functional classification system.

Lastly, the gait speed also had significant effect on the prediction accuracy especially for K3 subjects (Fig 4).

### Comparison with literature studies

As mentioned in the beginning, there are barely any published studies with the amputee population which makes a direct comparison of results difficult. Nevertheless, a comparison of the results of this study with the available literature is presented in Table 4.

**Table 4:** A comparison with the error magnitudes found in this study and the available relevant literature, ME: Mean Error, MAE: Mean Absolute Error.

Study	Subject population and task	Prediction method	HS error (ms)	TO error (ms)
This study	Amputee, N = 10, Level treadmill walking	Leg kinematics, Dual-minima of shank velocity	ME: -5.5 to -13.2, MAE: 17 to 41	ME: 34 to 81, MAE: 36 to 81
Maqbool et. al. 2015 [19]	Amputee, N = 1, Ramp ascent and descent	Leg kinematics, Dual-minima	ME: -37 to 13	ME: -17 to 122
Storm et al. 2016 [8]	Healthy, N = 10, Overground	Minima of shank velocity for HS. Acceleration-based for TO	MAE: 7 to 14	MAE: 16 to 51
Zahradka et al. 2020 [31]	Healthy and Gait-impaired, N = 17, Level treadmill walking	Minima of shank velocity for TO. Zero-crossing for HS	ME: -10.45	ME: -56.20
Trojaniello et. al. 2014 [16]	Healthy and Gait-impaired, N = 40, Overground	Minima of shank velocity for HS. Acceleration-based for TO	ME:0 to -22, MAE 10 to 22	ME: 0 to -16, MAE: 16 to 22
Lee & Park 2011 [23]	Healthy, N = 5, Overground	Leg kinematics, Dual-minima	ME: -17 to -21	ME: 3 to 15
Catalfamo et. al. 2010 [7]	Healthy and CP, N = 7, Overground and ramp	Leg kinematics, Dual-minima	ME:-8 to -21, MAE: 15 to 24	ME: 50 to 73, MAE: 50 to 73

Almost all studies have reported early TO prediction with this algorithm, albeit with smaller magnitudes than our results. Catalfamo et. al. [7] reported early TO prediction for all steps with a mean error of 50–73ms for healthy and children suffering from cerebral palsy. Trojaniello et. al. [16] reported mean absolute TO errors in the range of 16 to 22ms for elderly and gait-impaired subjects. These smaller magnitudes further reinforce our premise that the actual TO event occurs in the NP-ZC zone mentioned earlier for all populations.

Similarly, for HS prediction, the error values are smaller than for TO prediction as in this study. For instance, Zahradka et al. [31] reported a mean error of -10.45ms for a group of healthy and gait-impaired subjects which is very close to our results. Storm et. al. [8] reported absolute mean error for indoor and outdoor walking in healthy adults in the range of 11–14ms. Lastly, only two studies [8, 16] reported errors in temporal gait parameters exhibiting similar trend as our study (larger stance and swing time errors than step/stride errors). All in all, the findings of this study match well with the literature, exhibiting a higher level of accuracy for the HS prediction than for the TO prediction.

### Limitations

One limitation of this study is the use of treadmill walking data resulting in minimal gait variations. During overground walking, the amputee gait could present a more variations. This could make the prediction task more challenging for the algorithm leading to potentially larger errors than reported in this study. Moreover, only one out of 10 subjects was female, limiting the ability to study gender differences.

## Conclusion

To conclude, the lower-leg velocity signal can reasonably predict some of the gait parameters for amputee population, with statistically significant effect of leg, subject walking ability and gait speed on the prediction accuracy.

## Supporting information

S1 File Containing the data used to generate results in this article. (ZIP)
