# Supplementary material for: Estimation of gait parameters using leg velocity for amputee population
Source: PLoS One. 2022 May 13;17(5):e0266726. doi: 10.1371/journal.pone.0266726 (PMC9106160; doi:10.1371/journal.pone.0266726)
Supplement: S1 File — (ZIP) [file pone.0266726.s001.zip › DataShare/Readme 1.docx]

**Description of data file 'DataStruct.mat'**

The file ‘DataStruct.mat’ contains the event results available from the original data set (Hood et. al. 2020) as well as those generated by our the dual-minima algorithm. The file can be directly loaded into Matlab command window. It will create a variable named ‘ResultStruct’ in the Matlab workspace, which has 239 rows (1 for each trial) and 15 fields.

The data contained in these fields is detailed in the following table.

Abbreviations used:

1. Cont: Contralateral leg (intact leg)
2. Ipsi: Ipsilateral leg (prosthetic leg)
3. HS, EHS: Heel strike, Error in heel strike prediction
4. TO, ETO: Toe off, Error in toe off prediction
5. PF: Force Platform
6. Alg: Algorithm
7. tstamps: Time stamps

| **Sr. No.** | **Field name** | **Description** |
| --- | --- | --- |
|  | SubjectID | The subject identifier, the number followed by ‘TF’ in the original study (Hood et. al. 2020), also reproduced in Table 1 of this study |
|  | ContHStstampsPF | Timestamps of the event (HS or TO) reported in Hood et. al. 2020, based on the force platform data throughout the gait cycle.  The leg is either marked Contralateral or Ipsilateral (for intact vs. prosthetic leg, resp.) |
|  | ContTOtstampsPF |  |
|  | IpsiHStstampsPF |  |
|  | IpsiTOtstampsPF |  |
|  | ContHStstampsAlg1 | Timestamps of the event (HS or TO) calculated by the dual-minima algorithm throughout the gait cycle. |
|  | ContTOtstampsAlg1 |  |
|  | IpsiHStstampsAlg1 |  |
|  | IpsiTOtstampsAlg1 |  |
|  | ErrorEvents | A structure of the differences between corresponding timestamps from the force platform data and the algorithm, aka the prediction error. Contains four fields for both events and legs. |
|  | ProstSide | The amputated side of the subject (‘right’ or ‘left’) |
|  | Kgroup | The subject’s Medicare classification level as reported in Table 1 of the article |
|  | Age | The age of the subject |
|  | Gender | The gender of the subject (Male/Female) |
|  | WalkingSpeed | The walking speed during the trial [0.4 to 1.4m/s |
